# Integrating Traditional Breeding and Modern Biotechnology for Advanced Forest Tree Improvement

**DOI:** 10.3390/ijms26178591

**Published:** 2025-09-04

**Authors:** Zhongzheng Ma, Jingru Ren, Qianqian Liu, Jingjing Li, Haoqin Zhao, Dativa Gosbert Tibesigwa, Sophia Hydarry Matola, Tabeer Gulfam, Jingli Yang, Fude Wang

**Affiliations:** 1 State Key Laboratory of Tree Genetics and Breeding, Northeast Forestry University, Harbin 150040, China; 2 Institute of Forestry Science of Heilongjiang Province, Harbin 150081, China

**Keywords:** CRISPR/Cas, traditional breeding, MAS, GS, single nucleotide polymorphisms (SNPs)

## Abstract

In the context of global climate change and efforts toward “carbon peak and carbon neutrality,” forest resource protection and restoration have become fundamental to ecological civilization. The genetic improvement of trees, as the primary component of forest ecosystems, holds strategic importance for ecological security, resource supply, and carbon neutrality. Traditional tree breeding techniques, including selective and hybrid breeding, have established robust technical systems through extensive practice. However, these methods face limitations such as extended cycles, reduced efficiency, and constrained genetic gains in meeting contemporary requirements. Modern biotechnologies, including genomic selection (GS), gene editing (CRISPR/Cas9), and marker-assisted selection (MAS), substantially enhance the precision and efficiency of genetic improvement. Nevertheless, exclusive reliance on either traditional or modern methods proves insufficient for addressing complex environmental adaptation and rapid breeding requirements. Consequently, the integration of traditional breeding with modern biotechnology to develop intelligent, sustainable, and efficient breeding strategies has emerged as a central focus in tree genetics and breeding. An integrated “step-by-step” approach warrants promotion, supported by a multi-source data sharing platform, an optimized core germplasm repository, and a “climate-soil-genotype” matching model to facilitate the region-specific deployment of improved varieties.

## 1. Introduction

Forestry resources encompass the diverse resources and environmental conditions within forest ecosystems. These resources, being renewable, serve a vital function in maintaining ecological equilibrium and fostering socio-economic development [1,2]. The strategic significance of forestry resources has become increasingly evident as social productivity advances, accompanied by a marked increase in demand [3]. However, contemporary environmental and climatic changes pose unprecedented challenges to forestry resources. In response to severe environmental and climate change, the Chinese government has established objectives for carbon neutrality by 2030 and carbon peak by 2060 [4]. The protection and restoration of forestry resources have become critical priorities, as they represent key factors in achieving these “carbon peak” and “carbon neutrality” goals [5]. Within forest ecosystems, trees constitute the primary elements, serving not only as core components of forestry resources, but also as essential vehicles for achieving the “dual carbon” strategic objectives [6]. Consequently, the conservation and restoration of tree resources represent the primary focus of forestry resource protection, with tree genetic breeding providing crucial technical support and fundamental methodologies [7].

Tree genetic breeding represents a scientific discipline investigating forest genetics and the theory and technology of tree improvement guided by genetic principles. Its fundamental objective involves enhancing trees’ genetic characteristics, developing superior trait varieties, enabling environmental adaptation, strengthening survival capabilities, and facilitating adaptation to complex environments, thereby providing high-quality ecological environments and sustainable resources for human activities [8,9]. Conventional breeding requires substantial resources for managing and monitoring seed-generated families in nurseries and progeny tests [7].

Tree species breeding faces distinct challenges, including extended generation times and genomic complexity [10]. For many years, tree breeding primarily relied on traditional experiential knowledge without developing comprehensive scientific theories. The Industrial Revolution catalyzed significant improvements in productivity, leading to the gradual development of modern tree breeding research from the 19th century. Tree breeding practices received systematic guidance through the synthesis and application of scientific theories, and methodologies including artificial selection [11,12], hybrid breeding, and screening [13,14] progressively established a systematic scientific framework. Additionally, rapid industrialization has generated increased demand for wood processing products. Contemporary understanding of trees’ ecological functions has evolved, and the demand for high-quality tree species continues to grow, whether for resource utilization or ecological protection [15]. However, traditional breeding approaches encounter numerous limitations, including extended cycles, reduced efficiency, and hybrid incompatibility. Consequently, improving breeding efficiency and innovating breeding methods have become crucial considerations in modern tree breeding.

Since the advent of the 21st century, scientific and technological advancements have given rise to numerous innovative breeding techniques and theories. Notably, molecular breeding-based modern tree breeding techniques have substantially accelerated tree genetic breeding progress. Breeding methodologies, including genomic selection [16], gene editing [15,17], and ploidy breeding [18], in conjunction with modern biotechnology, have demonstrated significant impact in tree genetic breeding, emerging as crucial pathways for innovation in breeding. In the current era of rapid global economic and cultural development, maintaining equilibrium between wood utilization and ecological environment protection has become essential. While traditional breeding methods offer maturity, stability, and cost-effectiveness, their inherent limitations of low efficiency and extended cycles present challenges in meeting evolving breeding requirements. Conversely, molecular genetic breeding integrated with modern biotechnology offers considerable efficiency advantages, although its system remains in development and genetic transformation costs remain high. Consequently, the strategic integration of traditional breeding with modern biotechnology to develop breeding approaches suitable for contemporary needs has emerged as a critical challenge in modern tree genetic breeding.

## 2. Key Traditional Breeding Approaches for Genetic Improvement in Forest Trees

**Figure 1:**
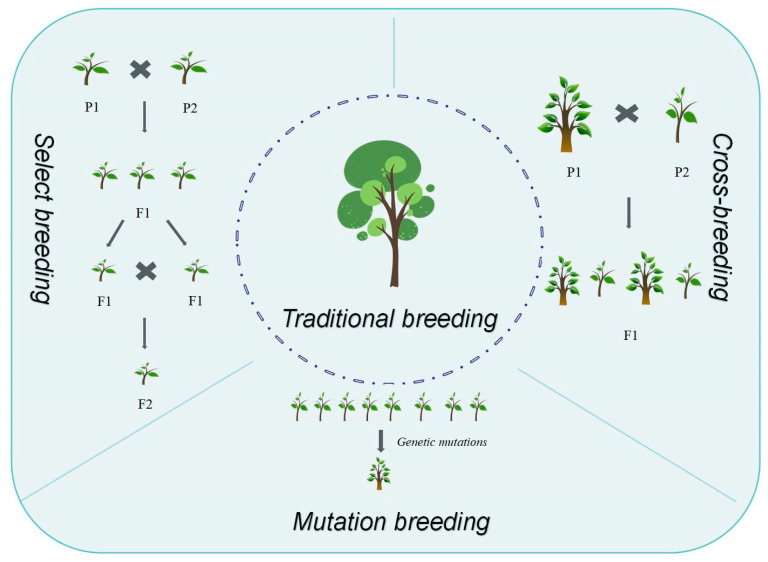
Traditional breeding methods and application.

Breeding represents a systematic process of developing improved varieties by selecting and cultivating superior strains from natural and artificially controlled variation populations to fulfill human requirements [19]. Tree breeding programs globally initiated primarily in the 1950 s, focusing on plus tree selection and progeny testing [20]. However, these early efforts relied predominantly on empirical knowledge without an established scientific theoretical framework. Modern forest tree genetic breeding emerged in the 19th century, leading to the maturation of conventional breeding methods [7,21]. Based on methodological distinctions, breeding approaches can be categorized into traditional breeding and biotechnological breeding [22,23]. Traditional breeding encompasses selective breeding, hybrid breeding, mutation breeding, polyploid breeding, and distant hybridization breeding (Figure 1). Selective breeding utilizes genetic variation and phenotypic traits as its foundation [24]. Its fundamental principle involves leveraging natural variation in forest trees, selecting individuals exhibiting desired traits for propagation, enabling trait accumulation and fixation in subsequent generations, thereby progressively enhancing tree genetic quality. This approach constitutes the cornerstone of forest tree genetic improvement. Hybrid breeding builds upon principles of genetic recombination and heterosis [25]. It encompasses controlled pollination between individuals with distinct genotypes, producing hybrid offspring with enhanced traits. Based on parental genetic distance, hybrid breeding encompasses both distant hybridization and inbreeding. Mutation breeding employs physical or chemical agents to induce genetic mutations or chromosomal variations within forest tree cells, generating novel genotypes [26,27].

## 3. Advantages in Adaptability, Accessibility, and Social Acceptance of Traditional Breeding

Traditional plant breeding methods have served as the foundation of agriculture, enabling the development of superior crop varieties with desirable characteristics. These methods have been refined over centuries of practice and evolved into a comprehensive and mature technical system [28]. Their extensive history and distinctive advantages remain irreplaceable. For example, in the 20th century, the IUFRO coordinated extensive international provenance trials across several Western European countries. In 1912, Henry successfully bred *P. generosa* through interspecies hybridization [29]. These techniques have demonstrated their validity through extensive practical application, with standardized operational processes based on natural genetic mechanisms, ensuring stable and controllable traits. This makes them highly suitable for large-scale implementation.

First, complex traits are typically controlled by multiple genes, and traditional breeding, through phenotypic integration and natural selection, effectively preserves synergistic gene combinations. This is particularly beneficial in enhancing environmental adaptability. For example, in European breeding programs for Norway spruce (*Picea abies*), superior families selected through over 20 years of systematic observation have demonstrated high growth adaptability even under significant climate change conditions [30]. Secondly, developing countries face several barriers to technological adoption: high acquisition costs, inadequate educational systems resulting in low technological literacy, and institutional weaknesses leading to ineffective policymaking. However, traditional breeding does not require sophisticated facilities like gene editing laboratories. It can be implemented through routine field management, making it cost-effective and appropriate for developing countries. For instance, Brazil’s Eucalyptus breeding program achieved yield increases exceeding 30% through hybrid seed production in seedling seed orchards, primarily utilizing parental selection and artificial pollination [31]. Furthermore, traditional breeding, based on hybridization and selection of natural germplasm resources, plays an essential role in maintaining species’ genetic diversity. Varieties developed through traditional breeding methods do not require rigorous genetically modified organism (GMO) safety reviews, resulting in reduced promotion resistance and greater social acceptance.

## 4. Limitations of Traditional Breeding

Traditional breeding, despite its advantages in technical maturity and cost-effectiveness for forest tree genetic improvement, encounters significant challenges regarding tree growth cycles, complex genetic backgrounds, and modern breeding requirements. These limitations become increasingly evident, particularly in the context of global climate change, frequent pest and disease outbreaks, and the demand for rapid breeding.

Long Breeding Cycles and Limited Genetic Gains: Most forest trees (e.g., pine, spruce) require 10–20 years to reach flowering and fruiting, resulting in a single breeding cycle lasting several decades. For example, Chinese fir (*Cunninghamia lanceolata*) achieved only a 35% genetic gain in volume after three generations of phenotypic selection (spanning 60 years), whereas molecular breeding could achieve similar results in one-third of the time [32]. Traditional breeding relies on phenotypic selection, making it difficult to rapidly accumulate genetic gains. Additionally, field trials are required to evaluate traits such as growth and stress resistance, which are time-consuming and susceptible to environmental interference, thus failing to meet the demand for rapid improvement.

High Dependency on Phenotypic Selection with Limited Accuracy: Phenotypes are significantly influenced by environmental and epigenetic factors, leading to selection bias during breeding. This manifests as environmental sensitivity and risks associated with subjective evaluation. Firstly, identical genotypes can exhibit substantial performance variations across different environments [33], including growth rate and physiological characteristics. Secondly, manual selection is susceptible to subjective influences, complicating the quantification and selection of complex traits.

High Randomness in Gene Recombination and Difficulty in Improving Target Traits: Traditional crossbreeding relies on natural gene recombination, where favorable alleles are frequently diluted or lost [34]. In crossbreeding, the linkage between target genes and undesirable genes is common, resulting in unfavorable traits in offspring.

Difficulty in Breaking Genetic Barriers and Limited Germplasm Innovation: Traditional breeding is primarily restricted to intra-species or closely related species hybridization, preventing the utilization of beneficial genes from distant species. Reproductive isolation exists among most forest trees, and hybrid incompatibility presents significant breeding challenges. Moreover, forest tree genetic resources are relatively limited. Although stress-resistant genes exist in wild relatives, traditional techniques alone are insufficient for incorporating these genes into cultivated varieties [35].

## 5. Advancements in Genomic Selection for Forest Trees: From Theory to Application

Genomic selection (GS) is a modern breeding technique that utilizes large marker sets with thousands of single nucleotide polymorphisms (SNPs) to estimate genomic breeding values. Its primary objective is to bypass phenotypic selection and directly identify superior genotypes, significantly reducing breeding cycles and enhancing genetic gain [16]. GS is characterized by three key aspects: First, it provides genome-wide coverage, enabling the analysis of hundreds of thousands to millions of markers, capturing genetic variations including minor-effect quantitative trait loci (QTLs) [36]. Second, GS employs a data-driven approach, utilizing machine learning and mixed linear models, in contrast to traditional empirical selection based on single traits [36]. Finally, GS facilitates cross-generational applications, enabling early selection of juvenile individuals [36].

GS operates within a fundamental framework, with its implementation comprising four distinct stages: The initial phase involves establishing a training population. The data requirements encompass multi-year, multi-location phenotypic data and high-density SNP chip sequencing-derived genotypes. Based on heritability calculations, the reference population typically requires more than 1000 individuals. The second phase focuses on model training and validation. Model prediction accuracy can be evaluated using K-Fold cross-validation, followed by selecting significantly associated SNPs or functional markers (such as promoter regions, non-synonymous mutations) for marker screening [37]. The third phase encompasses genomic estimated breeding Value (GEBV) prediction and application. genome-wide prediction (GP) results are commonly expressed as GEBVs. GEBVs can be calculated using genotypic data alone, substantially reducing field trial costs and streamlining candidate population screening. The final phase requires full-sib validation and breeding cycles. GEBV reliability can be verified through controlled pollination of offspring, and the model requires updates with new phenotypic data each generation to address genetic drift [38,39].

GS, through genome-wide marker-based prediction of breeding values, has emerged as a fundamental technology for genetic improvement in forest trees [40]. However, trees’ complex genomic characteristics (including high heterozygosity, polyploidy) and extended breeding cycles present distinct challenges to GS model accuracy. To accommodate tree genome complexity, such as marker prediction for polyploid trees (e.g., tetraploid poplar, hexaploid eucalyptus), traditional haplotype-based genome-wide prediction models require adaptation to multi-allelic dosage information prediction models [41]. Furthermore, environmental factors significantly influence genotype expression, particularly in climate-adaptive breeding, where genotype-environment interactions (GEI) warrant consideration. Incorporating environmental covariates (such as temperature, precipitation) into GBLUP models (G×EGBLUP), a method that identifies genome-wide SNPs subject to GEI, enhances the prediction accuracy of stress-resistant traits in trees.

## 6. Molecular Mechanisms of Gene Editing to Generate New Genotype

**Figure 2:**
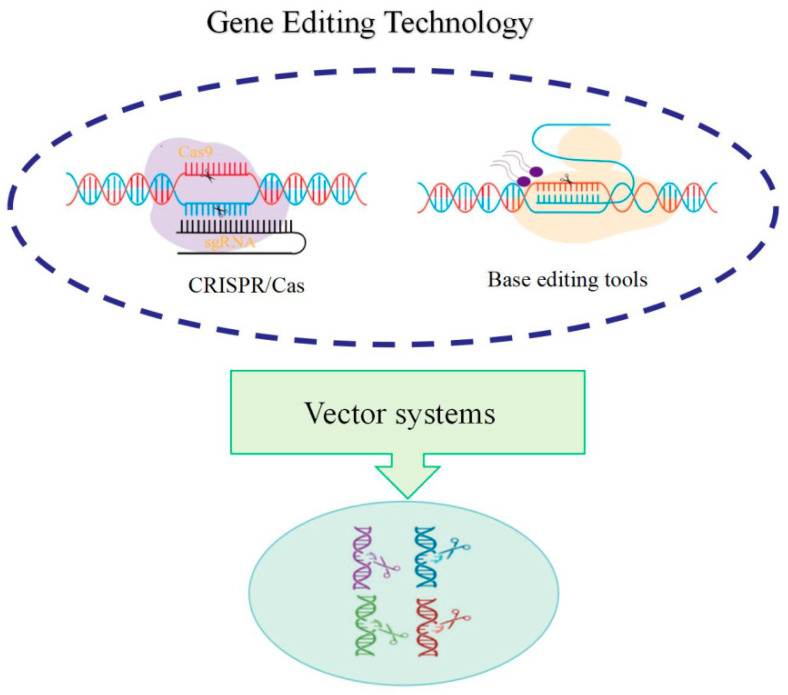
Molecular mechanisms of CRISPR/Cas9 system used to breed transgenic trees.

Gene editing enables targeting specific genomic locations through designed guide sequences, such as guide RNA in the CRISPR-Cas9 system, utilizing nucleases (such as the Cas9 protein) to create double-strand breaks (DSBs) at target sites. Cellular repair mechanisms (including non-homologous end joining, NHEJ, or homology-directed repair, HDR) repair the broken DNA, introducing specific genetic modifications during this process [42]. This technology presents several advantages: high targeting efficiency, straightforward design and operation, and cost-effectiveness, enabling precise forest tree genome modification. The CRISPR/Cas system demonstrates capability in simultaneous multiple gene knockout, substantially enhancing gene editing efficiency and effectiveness. Gene editing technology provides an essential tool for forest tree gene function validation. Through specific gene editing, researchers can observe and analyze gene functions, advancing understanding of molecular mechanisms underlying tree growth, development, and environmental adaptation. The CRISPR/Cas9 system (Figure 2) facilitates target gene knockout or mutation, enabling researchers to investigate specific gene roles in forest tree growth and development [43].

In the past five years, gene editing technology has experienced significant advancement, with particularly notable progress in its application to plants and forestry. The innovations and optimizations in gene editing technology, coupled with the development of new editors, have provided robust technical tools for genetic modification. Base editing and prime editing represent two crucial methodological approaches. CRISPR-based base editors (such as BE4max and ABE8e) achieve precise C → T or A → G substitutions without causing DNA double-strand breaks, thereby minimizing off-target effects. These technological innovations have facilitated breakthroughs in applying gene editing to forest tree genetic breeding [44]. Furthermore, gene editing technology enables complex trait regulation through multi-gene editing.

Gene editing technology has demonstrated considerable advantages in addressing forest tree regeneration and genetic stability challenges. Although Cas9 can be reprogrammed to target different genomic sites with relative ease, the on-target activity and off-target effects of individual sgRNAs exhibit substantial variation. Through optimization of sgRNA design and utilization of Cas9 variants, off-target effects can be minimized, ensuring precise and stable target gene editing. Moreover, researchers have developed a CRISPR/Cas12-based multi-target editing system to address the large genome characteristics of conifers, enabling simultaneous editing of multiple lignin synthesis-related genes and overcoming technical barriers posed by polyploidy. Additionally, the challenges of low regeneration rates require further investigation. Maybe morphogene-assisted transformation (MAT) could accelerate and improve plants’ transformation and regeneration.

## 7. Molecular Marker-Assisted Selection to Develop Target Traits

The main focus of Molecular marker-assisted selection (MAS) was on the search for QTLs, defined as regions of the genome associated with a particular phenotypic trait [45]. This approach expedites the selection of superior tree genotypes by screening molecular markers associated with target traits, while concurrently identifying molecular markers closely linked to desired characteristics (such as growth rate and stress resistance), thus enabling indirect selection of superior genotypes. Comparison of most widely used DNA marker system in plants was listed in Table 1.

**Figure 3:**
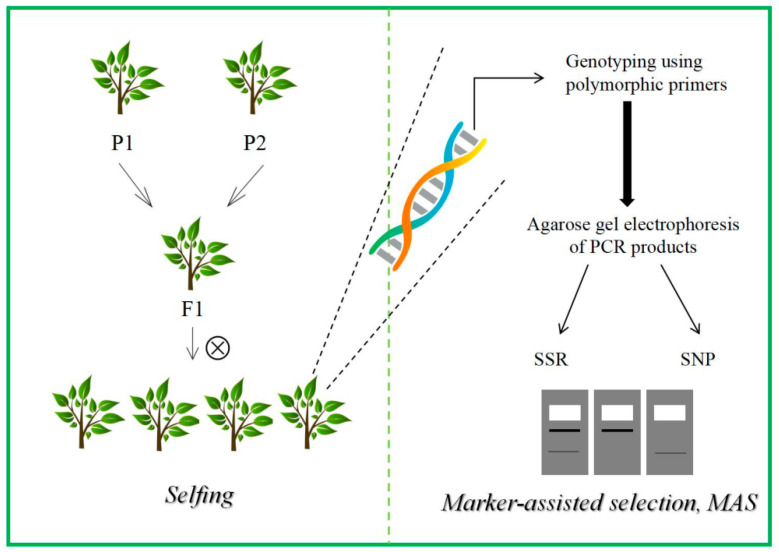
Schematic steps of marker-assisted selection.

The foundation of MAS rests on utilizing the linkage disequilibrium between markers and QTLs to overcome the time constraints of phenotypic observation, enabling efficient early selection. The fundamental process of molecular marker-assisted selection is illustrated in Figure 3. In comparison to conventional phenotypic selection, MAS provides distinct advantages including non-destructive testing, early-stage screening, and independence from environmental conditions [45]. The theoretical basis of MAS relies on the linkage relationship between genetic markers and target genes or traits, comprising three key components: linkage analysis, marker-trait association, and predictive model development. The implementation process consists of three primary steps (Figure 3):

Population Construction and Marker Development: Biparental mapping populations can be established through F2 or backcross populations, and high-throughput sequencing technologies enable the development of molecular markers associated with target traits. SNP chips developed using next-generation sequencing (NGS) can effectively cover functional regions of highly heterozygous genomes.

QTL Mapping and Validation: The integration of multi-environment phenotypic data, including drone remote sensing and LiDAR for obtaining three-dimensional phenotypes (tree height, crown width), minimizes environmental noise interference in QTL mapping.

Breeding Value Prediction and Selection: The approach combines genomic prediction models for genomic selection, incorporates genome-wide markers based on MAS, and employs machine learning to enhance prediction accuracy. While MAS focuses on major-effect QTLs, GS utilizes genome-wide markers to address minor-effect QTLs.

## 8. A Comprehensive Overview of Genome-Wide Association Studies (GWAS) in Complex Trait Analysis

GWAS is a genetic analysis method based on Linkage Disequilibrium (LD) [46]. It identifies genes or genomic regions associated with complex traits by detecting a large number of SNP markers across the entire genome. The fundamental principle of GWAS relies on linkage disequilibrium: within the genome, alleles at different gene loci combine non-randomly and exhibit specific associations. Through analysis of genotype data from numerous individuals, SNP markers associated with the target trait can be identified, enabling inference of the genes or genomic regions controlling the trait. Statistical methodologies, including General Linear Model (GLM) and Mixed Linear Model (MLM), facilitate association analysis between genotype and phenotype data, enabling identification of significantly associated SNP sites and subsequent candidate genes.

**Figure 4:**
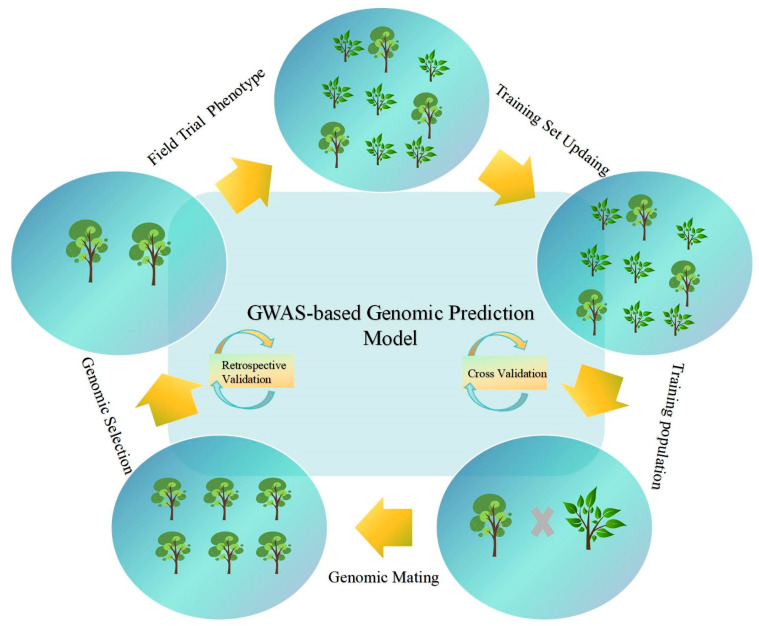
Flowchart of GWAS-based genomic prediction model optimization and breeding application.

The essential components of GWAS technology encompass population selection and phenotype identification [47]. The methodology requires selection of natural populations exhibiting rich genetic variation and broad genetic bases, incorporating germplasm resources from diverse geographical origins. Precise phenotypic measurement of target traits necessitates multi-year and multi-location repeated experiments to minimize environmental influences. Following phenotypic data collection, genotype identification proceeds using high-throughput SNP chips for population genotyping. After obtaining numerous SNP markers, whole-genome sequencing is conducted on the population individuals. Furthermore, population structure analysis employs Principal Component Analysis (PCA) to reduce population dimensionality and extract primary genetic structure information. The final phase involves screening candidate genes and conducting functional validation through techniques such as gene overexpression, RNA interference, or gene knockout to verify their role in the target trait. The fundamental workflow of GWAS analysis and applications is illustrated in Figure 4.

## 9. Integrating Traditional Breeding and Modern Biotechnology for Enhanced Forest Genetic Improvement

Traditional breeding and modern biotechnology each possess distinct advantages. An integration strategy combining these approaches is essential. Developing effective integration strategies for both methodologies is crucial for enhancing the efficiency and accuracy of forest genetic breeding. The strategy for integrating traditional breeding with modern biotechnology encompasses three primary aspects:

Integration of Traditional Breeding with MAS: Traditional breeding methods typically involve extended cycles, whereas MAS enables selection at seedling or early generation stages, facilitating early elimination of undesirable plants [48]. This approach reduces progeny population size, decreases workload, and shortens the breeding cycle. While traditional breeding faces challenges in combining multiple desirable traits, MAS facilitates the aggregation of multiple target genes into a single variety through molecular marker-assisted selection, thereby enhancing breeding efficiency. Selection accuracy represents a limitation of traditional breeding, where target genes may be linked with unfavorable genes, complicating separation during selection. MAS, founded on molecular biology principles, effectively prevents linkage drag and improves selection accuracy. Through early-stage screening, MAS eliminates undesirable plants efficiently, significantly enhancing selection effectiveness and supporting the development of superior varieties that align with market requirements [49].

GS with Phenotypic Data: The technical advantages of combining GS with phenotypic data manifest in three primary areas: enhanced prediction accuracy, transcendence of generational interval limitations, and elucidation of complex trait genetic structure. First, by integrating genomic data (SNP markers) with spatiotemporal dynamic phenotypes, GS captures gene-environment interaction (G × E) effects, substantially improving breeding value prediction accuracy and reducing model residuals [50]. Second, GS overcomes generational interval constraints. For instance, utilizing early hyperspectral imaging data in trees combined with GS models enables prediction of mature traits (20 years), significantly reducing the selection cycle. Additionally, the integration of GS with phenotypic data illuminates complex trait genetic structure. Through the combination of multi-time point phenotypic data with genomic data in longitudinal GS models, dynamic QTLs can be identified.

Gene Editing-Assisted Germplasm Innovation: The fundamental advantages of gene editing-assisted germplasm innovation include precise targeting to overcome genetic barriers, reduce breeding cycles, and surmount interspecies hybridization obstacles [51]. Traditional breeding faces inherent challenges including genetic barriers, extended breeding cycles, and interspecies hybridization limitations. Gene editing technology, through its precise targeting capabilities, effectively addresses these challenges. The technology enables targeted modification of specific genes, avoiding non-target trait linkage drag common in traditional hybridization. Furthermore, gene editing demonstrates significant advantages in gene insertion. Combined with hybridization techniques, it enables precise integration of desirable trait genes from distant species across the genome, overcoming interspecies hybridization barriers and expediting germplasm innovation.

## 10. Building an Efficient Forest Tree Breeding System for Sustainable Forestry

In response to global climate change pressures and forestry resource demands, establishing an efficient forest tree breeding system has become essential for sustainable forestry development. This system provides crucial guidance throughout the breeding process. Contemporary societal demands for forest tree genetic breeding encompass multiple dimensions, including ecological security, economic benefits, climate change adaptation, and sustainable development. Addressing these requirements necessitates constructing an efficient breeding system through three components: genetic resource conservation and technological innovation, elite breeding and promotion systems, and policy support and ecological security [52].

Genetic Resource Conservation and Technological Innovation: Genetic resources constitute the material foundation of forest tree breeding, with genetic diversity at its core. Systematic conservation and efficient utilization of germplasm resources are therefore paramount. A “three-step” strategy facilitates efficient resource utilization: Initially, establish a national forest tree germplasm resource map integrating satellite remote sensing and ground surveys. Subsequently, develop a core germplasm repository utilizing molecular marker technology to screen representative germplasm [53]. Finally, implement a germplasm resource sharing platform (comparable to the U.S. GRIN database) enabling integrated phenotype-genotype-environment data analysis. Technological innovation serves as the primary catalyst for overcoming traditional breeding limitations. Establishing an efficient forest tree breeding system requires accelerating the breeding process through advanced technologies, implementing modern biotechnologies including GS, gene editing, and molecular marker-assisted selection. Integration with traditional breeding methods enables practical application of breeding outcomes, surpassing previous limitations and expediting the breeding process.

Elite Breeding and Promotion System: Efficient breeding and precise promotion of elite varieties are essential for translating breeding achievements into practical outcomes. The breeding system optimization requires establishing a mother tree forest protection system to enhance high-quality seed yield and genetic quality; scientific configuration of seed orchards to improve seed genetic diversity and afforestation survival rates; and implementation of intelligent seedling cultivation technologies for production cycle monitoring and precise seedling growth control. Additionally, regionalized precise promotion necessitates developing a “climate-soil-genotype” matching model for location-specific breeding work. For instance, China’s regional layout of “southern eucalyptus and northern poplar” demonstrates southern eucalyptus clones achieving 8 m annual growth height, while northern poplar cold-resistant varieties survive in −30 °C environments, transcending traditional planting limitations.

Policy Support and Ecological Security: Institutional design and ecological risk management are fundamental for the sustainable operation of the breeding system. Establishing an efficient forest tree breeding system necessitates policy incentives and sustained investment mechanisms to facilitate breeding operations. Furthermore, breeding initiatives must address ecological security concerns through the implementation of prevention and control systems to ensure the sustainable development of forest tree genetic breeding. Moreover, international ecological protection objectives, such as “carbon neutrality,” require consideration in the selection and breeding of varieties that align with global ecological requirements.

In summary, developing an efficient forest tree breeding system should be founded on resource conservation, advanced by technological innovation, and reinforced by policy mechanisms, establishing a continuous cycle of “basic research-application transformation-ecological security.” Future endeavors should emphasize cross-disciplinary collaborations, including AI prediction models and international germplasm repository development, with the objective of achieving annual genetic gain growth exceeding 3% while providing intelligent solutions for global sustainable forestry development.

## 11. Challenges and Future Prospects

The integration of traditional breeding with molecular biology technologies presents significant challenges in multi-source data integration, particularly regarding technical data complexity. Cross-scale modeling of genomic, phenotypic, and environmental data requires sophisticated algorithms and substantial computational resources. Current statistical models demonstrate considerable limitations in analyzing multi-dimensional interaction effects (including gene-environment dynamic responses), thereby restricting prediction accuracy improvements. Additionally, modern biotechnology applications encounter technical constraints. The inefficiency of forest regeneration systems remains a significant impediment to gene editing, particularly evident in the low success rates of conifer somatic embryo regeneration, which hinders large-scale breeding of edited plants. Furthermore, polyploid forest trees’ gene redundancy characteristics complicate phenotypic breakthroughs through single-target editing, necessitating advanced multi-gene regulatory strategies.

A comprehensive regulatory framework for gene-edited forest trees remains undeveloped, particularly regarding classification standards for transgenic and exogenous DNA-free editing technologies. Regulatory disparities among nations impede international advancement. Additionally, excessive dependence on modern biotechnology may intensify genetic background simplification, potentially reducing forest populations’ resilience to pests, diseases, or abrupt climate changes, while also presenting ecological risks through gene flow dissemination. Consequently, current public perceptions regarding genome editing technology may influence the market integration of new species.

In 2018, Professor E. Buckler, a member of the U.S. National Academy of Sciences and a maize (*Zea mays*) genetic breeder, and others proposed the concept of intelligent design breeding, also known as the “Crop Breeding 4.0” era [54]. Its core idea is to achieve precise understanding and manipulation of crop genetic variation by integrating advanced genomics, information technology, and biotechnology, thereby more efficiently cultivating crop varieties adapted to modern agricultural environments, ensuring global food security and sustainable agricultural development. In future development directions, constructing an intelligent breeding technology system is crucial by developing interdisciplinary predictive models, integrating machine learning and environmental simulation tools, achieving dynamic optimization of breeding strategies, and simultaneously building standardized data sharing platforms to promote the global linkage analysis of genomic, phenotypic, and traditional breeding with modern biotechnology integration strategies and ecological environment data, enhancing the universality and iterative efficiency of models.

Current forest genetic breeding faces multifaceted issues, including genetic resource conservation, the complexity of breeding objectives, limitations of technical means, environmental change challenges, and socio-economic factors [55]. Considering the future development direction of the integration strategy of traditional breeding and modern biotechnology requires addressing these issues. The emergence of intelligent breeding development directions provides a good approach to solving these problems. Previously, the integration strategy of traditional breeding and modern biotechnology was discussed. On the innovative path of technological integration, a stepwise breeding process of “traditional selection—molecular markers—genome editing” can be constructed. Early stages involve marker-assisted screening of major genes, while middle and later stages combine gene editing for directional improvement of complex traits, thereby exploring the synergistic effects of epigenetic regulation and non-coding RNA in traditional hybridization, breaking through the limitations of relying solely on DNA sequence improvement. This aligns with the “Breeding 4.0 concept” proposed by Professor E. Buckler and others.

Therefore, in the future, the construction of an intelligent breeding technology system and the integration of traditional breeding with modern biotechnology will be important development directions for forest tree breeding. By developing interdisciplinary predictive models, building standardized data sharing platforms, constructing stepwise breeding processes, and exploring the synergistic effects of epigenetic regulation and non-coding RNA, the current challenges faced by forest genetic breeding, including genetic resource conservation, the complexity of breeding objectives, limitations of technical means, environmental change challenges, and socio-economic factors, can be effectively addressed.

## Figures and Tables

**Table 1:** DNA marker system in plants (Hasan et al., 2021) [45].

Feature and Description	RFLP	RAPD	AFLP	SSR	SNP
Genomic abundance	High	High	High	Moderate to high	Very high
Genomic coverage	Low copy coding	Whole genome	Whole genome region	Whole genome	Whole genome
Expression/inheritance	Co-dominant	Dominant	Dominant/co-dominant	Co-dominant	Co-dominant
Number of loci	Small (<1000)	Small (<1000)	Moderate (1000 s)	High (1000–10,000 s)	Very high (>100,000)
Level of polymorphism	Moderate	High	High	High	High
Type of polymorphism	Single base change, indel	Single base change, indel	Single base Change, indel	Changes In length repeat	Single base change, indel
Cloning and/or sequencing	Yes	No	No	Yes	Yes
Type of probes/primers	Low-copy DNA	10 bs random nucleotides	Specific sequence	Specific sequence	Allele-specificPCR
PCR-based	Usually no	Yes	Yes	Yes	Yes
Radioactive detection	Usually yes	No	Yes or no	Usually no	No
Reproducibility/reliability	High	Low	High	High	High
Amount of DNA required	Large (5–50 μg)	Small (0.01–0.1 Μg)	Moderate (0.51.0 μg)	Small (0.05–0.12 μg)	Small (>0.05 μg)
Genotyping throughput	Low	Low	High	High	High
Cost	Moderate to high	Low	Moderate	Moderate to high	High
Marker index	Low	Moderate	Moderate	Moderate to high	Moderate
Time demanding	High	Low	Moderate	Low	Low
Number of polymorphic per loci	1.0–3.0	1.5–5.0	20–100	1.0–3.0	1.0
Primary application	Genetic	Diversity	Diversity and genetic	All purposes	All purposes
